# Awareness and Practices of Menstrual Hygiene Among Rural Adolescent Schoolgirls in Lahore, Pakistan: A Cross-Sectional Study

**DOI:** 10.7759/cureus.73899

**Published:** 2024-11-18

**Authors:** Saba Shanza Afzaal, Shahzeb LNU, Sulaiman Tahir, Hamna Javed, Adil Saeed, Muhammad Haadi Ashal Pal, Maryyam Islam, Bilal Qammar

**Affiliations:** 1 Surgery, Lahore General Hospital, Lahore, PAK; 2 Obstetrics and Gynecology, Lahore General Hospital, Lahore, PAK; 3 Surgery, Punjab Rangers Teaching Hospital, Lahore, PAK; 4 Forensic Medicine, University Medical & Dental College, Faisalabad, PAK; 5 Internal Medicine, Jinnah Hospital, Lahore, PAK; 6 Internal Medicine, Aitchison College, Lahore, PAK; 7 Research and Innovation, Shalamar Medical and Dental College, Lahore, PAK; 8 Surgery, Shalamar Hospital, Lahore, PAK

**Keywords:** adolescent girls, child and adolescent, menstrual hygiene, research knowledge, self care practices

## Abstract

Background

Empowering adolescent girls with accurate data, a supportive environment, and access to resources is crucial for promoting their dignity and educational success, thereby enabling gender equity and social development in rural communities.

Aim

This research aims to assess the knowledge and practices of menstrual hygiene among adolescent girls in schools within the rural community of Lahore, Pakistan.

Methodology

This research employed a quantitative, cross-sectional design, with a survey conducted among 108 participants in Lakhodair Sharif Bakra Mandi, Lahore, Pakistan. Purposive sampling was used to select participants within the specified age range (e.g., 12-19 years old).

Results

The results indicate that 91 participants (84.25%) were aged 13-15 years, followed by 12 participants (11.11%) in the 16-18 age range. Four participants (3.70%) were aged 10-12 years, and only one participant (0.92%) was aged 19 or older. In terms of grade level, 71 participants (65.74%) were in grade 8, 29 participants (26.85%) were in grade 7, six participants (5.55%) were in grade 6, and two participants (1.85%) were in grade 5. Correlation analysis revealed a strong, positive correlation between menstrual hygiene knowledge and practice, with a correlation coefficient of 0.649 (p < 0.001).

Conclusions

This research found that individuals from rural areas of Lahore demonstrated a strong understanding of menstrual hygiene, reflecting high levels of knowledge. A positive relationship was observed between knowledge and hygiene practices. Additionally, the findings revealed that participants held positive attitudes toward menstrual hygiene, although they expressed concerns about abdominal pain, as well as challenges related to accessing facilities and ensuring privacy at schools.

## Introduction

Menstruation is a natural, physiological process that occurs monthly in women of reproductive age, characterized by hormonal fluctuations of estrogen and progesterone, and its primary function is the shedding of the uterine lining [1]. Studies have shown that menstrual hygiene management is a critical aspect of adolescent girls’ reproductive health, yet it is often overlooked, even in urban areas [2,3]. Although menstruation is a natural process, it is often surrounded by social stigma, misinformation, and inadequate access to necessary resources. Some studies [3,4] have noted that girls often lack the resources needed to manage their changing bodies effectively. In Pakistan, cultural taboos and social barriers, combined with limited access to hygienic products and education, result in poor menstrual hygiene practices. This is particularly true for adolescent schoolgirls in rural areas, who make up the majority of those affected [5].

The young girls of Lahore face challenges that are globally prevalent in terms of accessing menstrual hygiene knowledge and products [6]. The scarcity of clean water, sanitation, toilets, and menstrual sanitary products further exacerbates the struggles girls face during menstruation. Additionally, societal standards and taboos surrounding menstruation in some communities create an environment of ignorance and shame, preventing girls from seeking support or information [7]. Understanding the knowledge base and cultural practices related to menstrual hygiene among adolescent schoolgirls in rural Lahore is an important step toward addressing educational disparities, improving accessibility, and providing essential support [8]. By thoroughly analyzing the factors influencing menstrual hygiene management, targeted interventions can be implemented to promote health education, provide hygienic facilities, and challenge social norms around menstruation. Empowering adolescent girls with accurate information, a supportive environment, and access to resources is vital for promoting their dignity, educational success, and gender equity in rural communities [9]. In smaller communities in Lahore, menstrual hygiene issues are compounded by a lack of education, sanitary facilities, and menstrual protection products [10].

Aim and objectives

This research aims to identify the menstrual hygiene knowledge and practices among adolescent girls in schools within the rural community of Lahore, Pakistan. The objectives of the study are to assess the knowledge and practices related to menstrual hygiene among adolescent schoolgirls in rural Lahore, identify the barriers to practicing menstrual hygiene in these areas, and develop effective educational programs and interventions to improve menstrual hygiene practices while addressing the sociocultural taboos surrounding menstruation.

## Materials and methods

Study design

This research adopted a quantitative descriptive, cross-sectional design and was conducted at Shalamar Hospital, Lahore, Pakistan, from November 2023 to June 2024.

Study setting

The research was conducted at Lakhodair Sharif Bakra Mandi, Lahore, Pakistan, selected for its rural and semi-urban context. The study population consisted of high school girls primarily attending schools in Lakhodair Sharif Bakra Mandi. A random sampling technique was employed to select participants who met the eligibility criteria, ensuring effective representation of the target area in the study.

Sampling technique

The study focused on adolescent girls aged 12-19 years who reside in the ongoing population states of Lakhodair Sharif Bakra Mandi. Data were collected from 108 adolescent girls, with this sample size determined to account for potential complications during the data collection process and to ensure adequate representation of the entire study population.

Data collection procedure

Data were collected using a self-constructed questionnaire designed specifically for this study. The questionnaire, based on the Likert Scale, assessed menstrual hygiene knowledge, practices, access to resources, and demographic characteristics such as age, socioeconomic status, and household size. The questionnaire was validated by specialized doctors, and its reliability was checked using IBM SPSS Statistics for Windows, Version 29.0 (Released 2022; IBM Corp., Armonk, NY, USA), yielding a Cronbach’s alpha value of 0.78.

Data analysis

The responses gathered from the questionnaire were analyzed using IBM SPSS Statistics for Windows, Version 29.0. Descriptive statistics, including frequencies, percentages, averages, and standard deviations, were used to simplify and summarize the data.

Ethical considerations

The researchers obtained informed consent from all participants and ensured their anonymity and confidentiality during data collection, analysis, and reporting. Ethical guidelines set by relevant institutions and regulatory bodies were strictly followed throughout the study.

## Results

Table 1 presents the demographic information of the participants, including age, religion, mother’s educational level, grade, and father’s occupation. The age distribution of the sample group revealed that 91 participants (84.25%) were aged 13-15 years, followed by 12 participants (11.11%) in the 16-18 age range. About four participants (3.70%) were aged 10-12 years, with age showing the least variability (0.422), while the mother’s occupation exhibited the most variability. Only one participant (0.92%) was from a different age group. In terms of grade distribution, the majority of participants, 71 (65.74%), were in grade 8, followed by 29 (26.85%) in grade 7. A small number of participants, six (5.55%), were in grade 6, and two (1.85%) were in grade 5.

**Table 1:** Demographic statistics of participants

n = 108		N	Percentage (%)
Age (year)	10-12	4	3.7
	13-15	91	84.25
	16-18	12	11.11
	<18	1	0.92
Gender	Female	108	100
Religion	Muslim	105	97.22
	Non-Muslim	3	2.77
Grade	Grade 5	2	1.85
	Grade 6	6	5.55
	Grade 7	29	26.85
	Grade 8	71	65.74

The correlation analysis revealed a strong and direct positive correlation between knowledge and practice of menstrual hygiene, with a correlation coefficient of 0.649 (p < 0.001). This suggests that participants with higher levels of knowledge about menstruation are more likely to demonstrate positive practices related to menstrual hygiene. Thus, a significant connection exists between knowledge and adherence to recommended menstrual hygiene practices (Table 2).

**Table 2:** Correlation analysis of knowledge and practice among selected participants ** Positive correlation

Correlations		Knowledge of menstrual hygiene	Practice of menstrual hygiene
Knowledge	Pearson correlation	1	0.649^**^
	Sig. (two-tailed)		0
	N	108	108
Practice	Pearson correlation	0.649^**^	1
	Sig. (two-tailed)	0	-
	N	108	108

The model summary table indicates that the predictor variable, knowledge, explains a significant portion of the variance in the outcome variable, practice. The multiple regression analysis revealed that knowledge accounts for approximately 42.2% of the variance in practice, highlighting its direct and significant impact on menstrual hygiene practices. The F-test (F = 77.336, p < 0.001) further confirms the statistical significance of the model in predicting practices based on knowledge (Table 3).

**Table 3:** Model summary for participants

Model summary							
Model	R	R square	Adjusted R square	Standard error of the estimate	Change statistics		
					R square change	F change	p-value
1	0.649	0.422	0.416	0.32329	0.422	77.336	<0.001

The ANOVA table highlights that the regression model is significant in predicting the practice variable based on knowledge. The F-statistic (F = 77.336, p < 0.001) (Table 3) indicates that variations in the knowledge scores are significantly associated with differences in the practice scores, as shown in Table 4.

**Table 4:** Analysis of questionnaire responses

Question	Strongly disagree	Disagree	Neutral	Agree	Strongly agree	Total
You heard about periods before menarche.	2	-	16	67	23	108
You get information about periods from your mother.	-	2	27	52	27	108
You get information about periods from your class teacher.	5	-	13	63	27	108
You get information about periods from your friends or relatives.	2	2	9	48	47	108
You know about the normal duration of the menstrual cycle.	2	-	16	67	23	108
You know about the normal menstrual bleeding duration.	2	-	27	52	27	108
You have abdominal pain during periods.	2	2	9	47	48	108
You know that menstruation is a lifelong process.	3	-	18	65	22	108
You record the date of periods.	2	2	9	50	45	108
You use cloth as an absorbent during periods.	2	2	9	48	47	108
You face privacy issues when washing your (cloth) absorbent.	2	2	9	48	47	108
You use sanitary pads as absorbent during periods.	2	-	16	67	23	108
Two to three sanitary pads changed in a day during periods.	3	2	9	48	46	108
You bunked school during periods due to pain and vomiting.	2	2	9	48	47	108
You bunked school during periods due to the lack of a toilet facility at your school.	2	2	26	48	30	108

## Discussion

This research found that people from Lakhodair Sharif Pura Bakra Mandi in Lahore demonstrated a strong understanding of menstrual hygiene due to their high level of knowledge. A positive relationship was observed between knowledge and hygiene practices. However, despite these positive attitudes toward menstrual hygiene, participants expressed concerns regarding abdominal pain and challenges accessing facilities and privacy at schools [11]. These findings align with Chandra-Mouli and Patel’s study [12], where 58% of adolescent girls lacked knowledge about menarche before its onset. Kapoor and Kumar’s study [13] also noted that mothers were the primary informants for 71.33% of the girls, followed by their sisters at 23.78%. Menarche, an important milestone in adolescence, is ideally understood through guidance from mothers. The low level of understanding in this rural area can be attributed to the generally low education levels of female students [14].

In Nandraj and Duggal’s research, 59.09% of girls exclusively used sanitary pads, while 40.91% relied on old or new cloth material, either alone or in combination with sanitary pads. In this study, eight out of 10 girls used sanitary pads only, while the remaining two girls used cloth material, either new or old, alone or with pads. Despite 83.33% of respondents handling worn material and underwear, only 13.64% faced challenges in washing or drying due to lack of space, water, or privacy [15]. These findings are consistent with Bloomfield’s study, which reported that 15.7% of girls used old washed cloth, and 70% faced issues with washing, drying, and privacy [14]. Additionally, a shortage of water was identified as a significant issue contributing to improper washing of cloth [16]. Non-affordability of pads due to high costs was another major barrier to using sanitary products [17]. Sadiq and Salih also highlighted the importance of bathing during menstruation, with 86.08% of girls agreeing that daily bathing is essential. Washing hands with water and soap was a common practice among the girls [18].

This study also found that concerns about privacy in schools hindered the ability to follow proper hygiene practices. However, there are some limitations to this research. The sample size is small, limiting the ability to generalize the findings. Additionally, the study is geographically specific to areas in Lahore, making it difficult to apply the findings universally. Nonetheless, the research provides valuable insights into the menstrual hygiene knowledge and practices of adolescent girls in rural Lahore, highlighting the need for improved access to resources, privacy, and education.

## Conclusions

This research provided valuable insights into the demographic characteristics, knowledge, and awareness of menstrual hygiene among the surveyed participants. The majority of participants were aged 13-15 years and identified as Muslims. Descriptive statistics revealed that the participants had a strong understanding of menstrual hygiene, as evidenced by their high knowledge scores and hygiene practices. The correlation analysis confirmed a significant positive relationship between hygiene practices and knowledge, indicating that greater knowledge is associated with better adherence to recommended hygiene practices. The ANOVA results and regression analysis further supported these findings, showing that knowledge was a key predictor of menstrual hygiene practices. While the participants demonstrated positive attitudes toward menstrual hygiene, they expressed concerns about abdominal pain and the lack of access to proper facilities and privacy at schools.
